# Influence of Differ P Enrichment Frequency on Plant Growth and Plant C:N:P in a P-Limited Subtropical Lake Wetland, China

**DOI:** 10.3389/fpls.2018.01608

**Published:** 2018-11-05

**Authors:** Feng Li, Cong Hu, Yonghong Xie, Wenzhi Liu, Xinsheng Chen, Zhengmiao Deng, Zhiyong Hou

**Affiliations:** ^1^Key Laboratory of Agro-ecological Processes in Subtropical Region, Chinese Academy of Sciences, Changsha, China; ^2^Dongting Lake Station for Wetland Ecosystem Research, Institute of Subtropical Agriculture Chinese Academy of Sciences, Changsha, China; ^3^Key Laboratory of Aquatic Botany and Watershed Ecology, Chinese Academy of Sciences, Wuhan Botanical Garden, Wuhan, China

**Keywords:** *Carex brevicuspis*, plant C:N:P stoichiometry, P enrichment frequency, biomass accumulation, biomass allocation

## Abstract

Phosphorus (P) enrichment as a result of anthropogenic activities can potentially alter plant C:N:P stoichiometry. However, the influence of different P enrichment frequencies on plant C:N:P stoichiometry in P-limited ecosystems is still unclear. In this study, we conducted a P-addition experiment to elucidate the effect of various P enrichment frequencies on the plant C:N:P stoichiometry of *Carex brevicuspis* in a freshwater wetland at Dongting Lake, China. We used four P enrichment frequencies (treatment A: no P addition; treatment B: three 0.1 g kg^−1^ additions at 10-day intervals; treatment C: two 0.15 g kg^−1^ additions at 15-day intervals; and treatment D: one 0.3 g kg^−1^ addition during the experimental period) in a factorial design with an experimental duration of 30 days. Biomass accumulation was lowest in the treatment A and highest in the treatment C, and increased with decreasing P addition frequency. The shoot:root ratio did not differ significantly between the four treatments. Both foliar and root C concentrations were not significantly different between the treatments. Foliar N concentration was significantly lower in the treatment D than in the other three treatments, while root N concentration did not differ significantly between the treatments. Both foliar and root P concentrations, and foliar C:N were much higher in the treatment B than in the treatment A. However, root C:N did not differ significantly between treatments. Both foliar and root C:P and N:P of *C. brevicuspis* were lower in the treatment B than in the treatment A. These results indicated that different frequencies of P addition significantly influenced plant growth. Moreover, P enrichment, rather than frequency, significantly influenced plant C:N:P stoichiometry. Our results improve our understanding of the influence of different P enrichment frequencies on plant C:N:P stoichiometry and nutrient cycling in freshwater wetlands.

## Introduction

Phosphorus (P) plays an important role in determining plant growth and community structure in both terrestrial and aquatic ecosystems (Elser et al., 2007; He and Dijkstra, 2015; Mao et al., 2016). Recently, anthropogenic discharges of P have doubled the natural P loading due to the intensity of activities such as aquaculture and agriculture (Mao et al., 2015). These large quantities of P are discharged into wetland ecosystems, leading to a series of ecological and environmental problems, such as eutrophication and biodiversity reduction (Rashid and Romshoo, 2013; Bu et al., 2016).

Relative abundances of carbon (C), nitrogen (N), and P, and C:N:P stoichiometry in plants are powerful indicators of ecological processes, e.g., community organization, nutrient limitation, food webs, and decomposition (Elser et al., 2000; Güsewell et al., 2003; Xia et al., 2014). Therefore, studies on plant ecological stoichiometry enhance our understanding of the growth and nutrient-use strategies of plants, and their responses to various environmental stresses. For example, plant C:N:P stoichiometry is significantly affected by external nutrient availability (Elser et al., 2007; Mao et al., 2016). To date, the influence of increased P loading on plant C:N:P stoichiometry has been studied in various wetland ecosystems (Rejmánková et al., 2008; Mao et al., 2016). Most of these studies confirmed that increasing P loading enhanced plant P concentration and decreased C:P, while results regarding the influence of increasing P loading on plant N concentration, C:N, and N:P were inconsistent across studies (Mao et al., 2016). In P-limited ecosystems, P enrichment would promote plant growth and reduce plant N concentrations and N:P ratios, mainly due to the dilution effect (Feller et al., 2007; Mao et al., 2016). However, in N-limited ecosystems, the response of plant N concentration, C:N ratio and N:P ratio might be determined by the plant’s nutrient use strategies (Yuan and Chen, 2015; Mao et al., 2016). Therefore, more studies are needed to investigate the general influence of P enrichment on plant C:N:P stoichiometry.

Nutrient concentrations can increase at different rates over different spatiotemporal scales, which influences plant growth performance (Xie et al., 2004; Zhang et al., 2018). For instance, biomass allocation patterns and P allocation ratios in *Eichhornia crassipes* differed significantly with different modes of nutrient increase (Xie et al., 2004). Different nutrient enrichment rates would alter soil nitrogen-phosphorus imbalances, affecting the structure, function, and diversity of ecosystems and organisms (Peñuelas et al., 2013). Sun et al. (2018) also confirmed that different N and P input ratios significantly changed the levels of N, P, and other elements in plants, and that the influence differed among different plant organs. While many studies have investigated the influence of P enrichment on plant C:N:P stoichiometry in different types of wetlands (Newman et al., 2004; Feller et al., 2007; Mao et al., 2016), the effects of different P enrichment frequencies on plant stoichiometry remain unclear.

This study investigated the effects of different P enrichment frequencies on wetland plant stoichiometry at Dongting Lake wetland, China. This lake is the second largest freshwater lake in China and has the largest water exchange capacity with the Yangtze River (Xie and Chen, 2008). Our previous studies confirmed that plants were limited by P in this lake (Li et al., 2017, 2018), but it has received increasing inputs of P mainly due to the use of agricultural fertilizer in the local area. The quantity of P input into the lake is about 4.1 × 10^4^ t annually (He et al., 2009). Here, we report the changes in plant C:N:P stoichiometry and growth performance of *Carex brevicuspis* after four different P enrichment frequency treatments. Based on the above statement, we hypothesized that (1) P enrichment would promote the growth of *C. brevicuspis* and the influence differ with different P enrichment treatments; (2) P enrichment would increase plant P concentration and C:N ratio, but reduce plant N concentration, C:P and N:P ratios. Moreover, these influences would differ with different P enrichment frequencies.

## Materials and Methods

### Study Site

**FIGURE 1:**
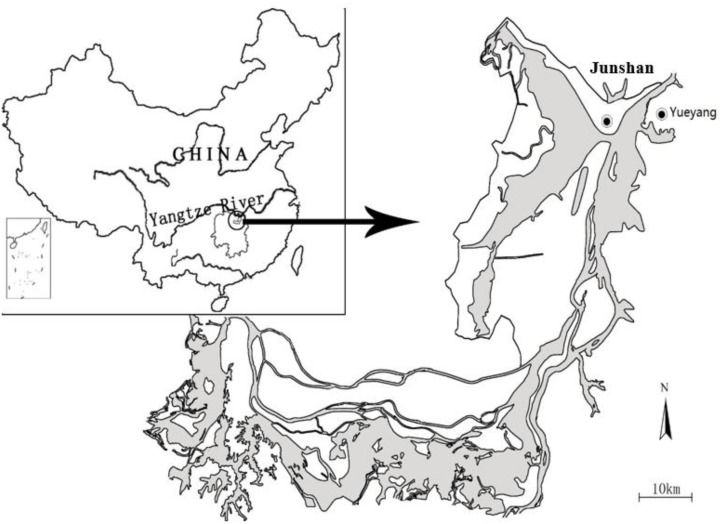
Dongting Lake, showing the location of the study site. The shaded areas represent the wetlands.

Dongting Lake (28°30′–30°20′ N, 111°40′–113°10′ E) is located on the south bank of the mid-section of the Yangtze River (Xie and Chen, 2008). The lake receives inflow from four rivers (Xiang, Zi, Yuan, and Li) in Hunan Province and four channels (Songzikou, Taipingkou, Ouchikou, and Tiaoxiankou) connect it to the Yangtze River (Figure 1). The wetlands are characterized by large seasonal fluctuations in water level and are usually completely flooded from May to October and susceptible to drought from November to April. The mean annual temperature is 16.8°C, with hot summers (June to August, 27.3°C) and cold winters (December to February, 5.8°C). Annual precipitation is 1382 mm, with more than 60% falling from April to August (Deng et al., 2015).

### Study Species

*Carex brevicuspis* is widely distributed in Taiwan and eastern mainland China (Dai et al., 2010). At Dongting Lake, this species can form mono-dominant communities or co-exist with other species e.g., *Miscanthus sacchariflorus* and *Polygonum hydropiper*. *C. brevicuspis* usually has two growing phases which are related to changes in the water rhythm in Dongting Lake, usually flowering and fruiting in April or May before the flooding, and remaining completely submerged during the flooding season. After flooding, the shoots emerge immediately (November) and grow until January. In January, the above-ground plant parts wither. Subsequently, new ramets emerge and grow rapidly in February and/or March (Deng et al., 2015). In the Dongting Lake, the *C. brevicuspis* community plays an important role in biodiversity maintenance owing to its multiple ecological functions, for example as a food resource for migratory birds and a spawning ground for migratory fish.

Plants were collected in May 2017 from Junshan county (29°22′ N, 112°59′ E), East Dongting Lake wetland. Small blocks (25 × 25 cm) of *C. brevicuspis* vegetation were cut and transported to an experimental field at the Dongting Lake Station for Wetland Ecosystem Research, Chinese Academy of Sciences. The blocks were placed in plastic buckets (87 × 65 × 62 cm), which contained 20 cm soil (7.07 mg g^−1^ organic matter, 0.87 mg g^−1^ total N, and 0.73 mg g^−1^ total P; Table 1) to germinate new ramets. Soil was also collected from the area (0–20 cm depth) in which the *C. brevicuspis* plants were collected. The plants were watered once weekly with tap water (0.511 μg L^−1^ NH_4_^+^−N, 1.760 μg L^−1^ NO_3_−N, 0.527 μg L^−1^ PO_4_^3+^−P, pH = 7.2).

**Table 1:** Soil characteristics (mean ± SE) after different P addition frequency treatments (treatments A–D represent: no P addition treatment; three – time P addition treatment; two-time P addition treatment; and one-time P addition treatment, respectively).

Treatments	Total nitrogen content (mg g^−1^)	Total phosphorus content (mg g^−1^)	Organic carbon content (mg g^−1^)	C:N	C:P	N:P
A	0.87 ± 0.02^a^	0.73 ± 0.01^b^	7.07 ± 0.14	8.20 ± 0.14^b^	9.69 ± 0.15	1.18 ± 0.02^a^
B	0.82 ± 0.02^ab^	0.77 ± 0.01^ab^	7.23 ± 0.15	8.87 ± 0.14^ab^	9.42 ± 0.17	1.07 ± 0.03^ab^
C	0.79 ± 0.05^ab^	0.79 ± 0.02^a^	7.14 ± 0.25	9.25 ± 0.42^a^	9.11 ± 0.34	1.00 ± 0.06^bc^
D	0.72 ± 0.04^b^	0.77 ± 0.02^ab^	6.87 ± 0.24	9.64 ± 0.25^a^	8.92 ± 0.33	0.94 ± 0.05^c^

*Different letters indicate significant differences among treatments at the 0.05 significance level.*

### Experimental Design

Four P enrichment treatments were applied: treatment A – no P addition; treatment B – three 0.1 g kg^−1^ P additions at 10-day intervals; treatment C – two 0.15 g kg^−1^ P additions at 15-day intervals; and treatment D – one 0.3 g kg^−1^ P addition during the experimental period.

On July 11, 2017, 840 seedlings of similar size (3–4 leaves, 20 ± 3 cm in height) were transplanted into 56 pots (30 cm height, 23 cm diameter, and 15 seedlings per pot), which were filled with 7 kg soil (same soil as for seedling cultivation). All pots were placed into 1 of 7 cement ponds (100 × 100 × 100 cm, two pots per treatment per pond) in a random block design. Experimental treatments began on July 18, 2017. Phosphorus was added as NaH_2_PO_4_. Firstly, the required mass of NaH_2_PO_4_ was dissolved in 150 ml tap water and then sprayed uniformly into the pot. For each P addition treatment, the pots that did not receive NaH_2_PO_4_ were leached using 150 ml tap water. Each pot was supplied 500 mL tap water once weekly and exposed to natural sunlight.

### Harvest

The plants were harvested 30 days after the first P treatment, before they flowered. Plant roots were carefully dug out by hand and rinsed using tap water to remove sediment. Then, plant parts were separated into leaves and roots (root and rhizome), due to the rhizome is difficult to separated. All parts were oven dried at 80°C for 48 h and weighed. Biomass accumulation was calculated as the collective mass of all tissues. Shoot:root ratio was defined as the ratio of leaf mass to root mass. After plant harvest, we collected soil samples at each pot for soil analysis.

### Laboratory Analysis

Dry foliar and root samples were ground for further analysis. Total N and C concentrations were measured using an elemental analyzer (Vario MAX CN, Elementar, Germany), and total P concentration was measured using the molybdenum blue colorimetric method after digesting the samples in a solution of H_2_SO_4_ and H_2_O_2_ (Zhang et al., 2015).

Soil samples were air-dried and sieved through a 0.15 mm sieve before analysis. Soil organic C content was measured using wet oxidation of organic matter with a solution of KCr_2_O_7_ and H_2_SO_4_, followed by back-titration using FeSO_4_. Soil N concentration was measured using the Kjeldahl method and soil P concentration was measured using acid digestion with a solution of H_2_SO_4_ and HClO_4_ (Zhang et al., 2015).

### Data Analysis

One-way analysis of variance (ANOVA) was performed in conjunction with Duncan’s test to determine the effect of P addition frequency on biomass accumulation, shoot:root ratio, and plant stoichiometry of *C. brevicuspis*, as well as soil characteristics (total N, total P, organic carbon content, C:N, C:P, and N : P). Tukey’s *post hoc* tests were used for multiple comparisons. Data were log10-transformed where necessary to reduce the heterogeneity of variance. Liljefor’s test and Levene’s test were used to test the normality and homogeneity of data, respectively. All statistical analyses were conducted using SPSS ver. 15.0 (SPSS Inc., Chicago, IL, United States).

## Results

### Biomass Accumulation and Shoot:Root Ratio

**FIGURE 2:**
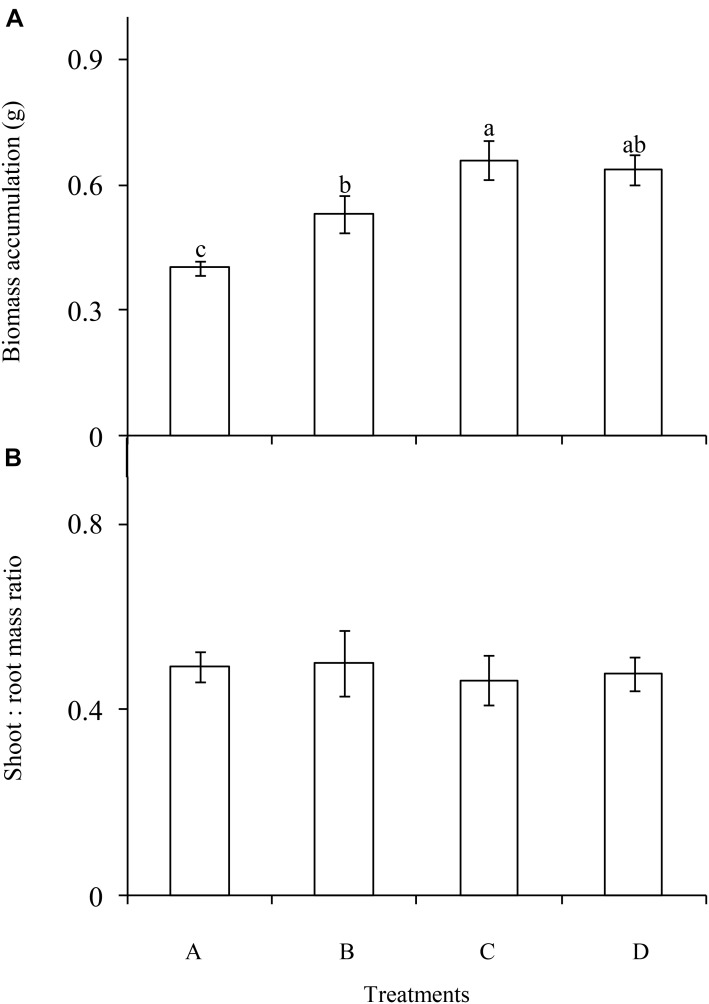
Biomass accumulation **(A)** and allocation **(B)** of *Carex brevicuspis* (means ± standard errors, *n* = 7) under different P addition frequencies (treatments A–D represent: no P addition treatment; three - time P addition treatment; two-time P addition treatment; and one-time P addition treatment, respectively). Different letters indicate significant differences between treatments at the 0.05 significance level.

The P enrichment frequency had a significant influence on the biomass accumulation of *C. brevicuspis* (*F* = 9.562; df = 6; and *P* < 0.001; Figure 2A), which was lowest in the treatment A and highest in the treatment C. Moreover, biomass accumulation increased with decreasing P enrichment frequency. In contrast, shoot:root ratio did not differ significantly between the four frequency treatments (*F* = 0.103; df = 6; and *P* > 0.05; Figure 2B).

### Foliar Stoichiometry

**FIGURE 3:**
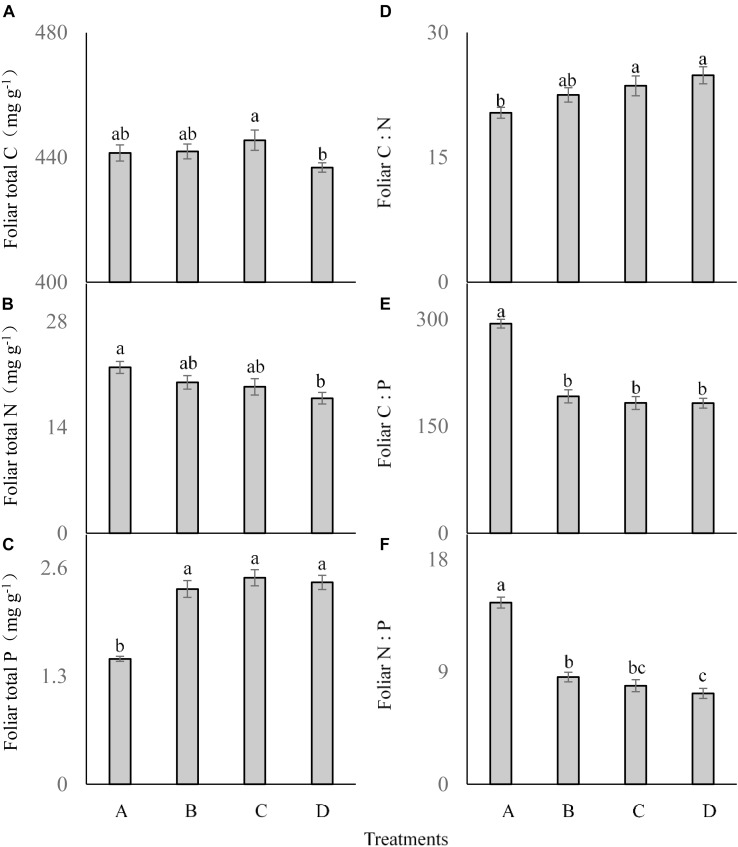
Foliar ecological stoichiometry **(A–F)** of *Carex brevicuspis* (means ± standard errors, *n* = 7) under different P addition frequencies (treatments A–D represent: no P addition treatment; three - time P addition treatment; two-time P addition treatment; and one-time P addition treatment, respectively). Different letters indicate significant differences between treatments at the 0.05 significance level.

The frequency of P addition had no significant influence on the foliar total C concentration of *C. br*e*vicuspis* (*F* = 2.041; df = 6; and *P* > 0.05; Figure 3A), although it was lower in the treatment D than in the other treatments. Foliar total N was significantly affected by P addition frequency (*F* = 3.465; df = 6; and *P* < 0.05; Figure 3B), which was much lower in the treatment D than in the other three treatments. Foliar total P concentration was much higher in the three P addition treatments than in the control (*F* = 30.219; df = 6; and *P* < 0.05; Figure 3C), but did not differ significantly between the three P addition treatments.

Foliar C:N showed a similar trend as foliar P concentration, which was much higher in the three addition treatments than in the control (*F* = 4.009; df = 6; and *P* < 0.05; Figure 3D), but did not differ significantly among the three P addition treatments. Both foliar C:P (*F* = 47.787; df = 6; and *P* < 0.05; Figure 3E) and foliar N:P (*F* = 61.053; df = 6; and *P* < 0.05; Figure 3F) showed similar trends, and were much lower in the three P addition treatments than in the control.

### Root Stoichiometry

**FIGURE 4:**
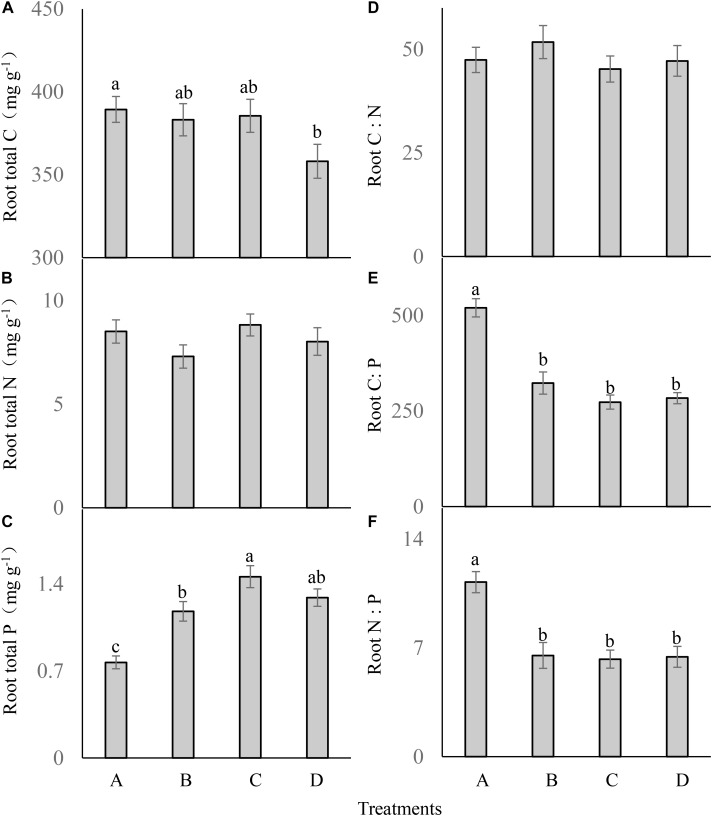
Root ecological stoichiometry **(A–F)** of *Carex brevicuspis* (means ± standard errors, *n* = 7) under different P addition frequencies (treatments A–D represent: no P addition treatment; three - time P addition treatment; two-time P addition treatment; and one-time P addition treatment, respectively). Different letters indicate significant differences between treatments at the 0.05 significance level.

Root total C (*F* = 2.223; df = 6; and *P* > 0.05; Figure 4A) and total N (*F* = 1.299; df = 6; and *P* > 0.05; Figure 4B) were not significantly different between the treatments. Different P addition treatments significantly influenced the root P content (*F* = 15.931; df = 6; and *P* < 0.05; Figure 4C), which was much higher in the three P addition treatments than in the control, and the highest root P content occurred in the treatment C.

Root C:N did not differ significantly between the treatments (*F* = 0.541; df = 6; and *P* > 0.05; Figure 4D). Both root C:P (*F* = 31.954; df = 6; and *P* < 0.05; Figure 4E) and N:P (*F* = 12.001; df = 6; and *P* < 0.05; Figure 4F) were significantly lower in the three P addition treatments than in the control, but were not significantly different between the three P addition treatments.

## Discussion

Our study confirmed that biomass accumulation in *C. brevicuspis* was much higher in the three P addition treatments than in the control, and much higher in the two-time P addition treatment than in the three-time P addition treatment. These results were consistent with hypothesis 1 and suggested that both P addition and addition frequency significantly influenced growth of *C. brevicuspis*. Moreover, our results confirmed that P enrichment frequency did not significantly influence the biomass allocation of *C. brevicuspis*, indicating that this was not an effective way for plants to acclimate to different P enrichment frequencies.

The stimulation of plant growth by additional P has been widely reported in other studies (Chiang et al., 2000; McCormick et al., 2001; Mao et al., 2016). For instance, addition of P resulted in a two-fold increase in the biomass of sawgrass and mixed sawgrass-cattail communities in the Everglades Wetland, United States (Chiang et al., 2000). Studies have also confirmed that water lily leaf size was enhanced in response to P enrichment (McCormick et al., 2001; Newman et al., 2004). However, our results contradict those of some other studies (Song et al., 2011; Gao et al., 2016). For instance, Song et al. (2011) found that increased P input had no effect on aboveground biomass of *Calamagrostis angustifolia* in the Sanjiang Plain Wetland, China, which might have been because plants were adapted to earlier soil P conditions and responded slowly to the addition of P (Macek and Rejmánková, 2007). Another possibility is that P was not a limiting nutrient in the Sanjiang Plain Wetland (Mao et al., 2016). However, our previous study showed that *C. brevicuspis* was limited by P at Dongting Lake (Li et al., 2017, 2018); this might explain why the addition of P promoted *C. brevicuspis* growth.

Both foliar and root P concentrations in *C. brevicuspis* were significantly increased in the three P addition treatments, which might explain the decrease in C:P and N:P in these treatments. These results were partially consistent with our hypothesis 2. Increased plant P concentration following P enrichment has been widely reported (Ostertag, 2010; Song et al., 2011; Mao et al., 2016). However, study of *Eleocharis* spp. showed that leaf tissue P content decreased with the addition of P, and caused higher C:P and N:P in enriched plots (Daoust and Childers, 2004). These results indicate that plant P concentration responses to P enrichment are species-specific and might be related to plant absorption efficiency and nutrient use strategies, as well as soil microorganism and enzyme activity (Rejmánková et al., 2008; Song et al., 2011; Yuan and Chen, 2015). Moreover, our study confirmed that both foliar and root P concentrations, C:P, and N:P in *C. brevicuspis* were not significantly different between the three P addition treatments. However, foliar N:P and root P concentration were significantly different, indi**c**ating that the influence of P addition frequency on plant stoichiometry was relatively weak. These results contradict our hypothesis 2 and the findings of other studies (Sun et al., 2018). A possible reason is that the amount of P in the three–phase frequency addition treatment was sufficient to support the growth of *C. brevicuspis*. Another possible reason is that *C. brevicuspis* has a relatively high stoichiometric homeostasis, which may have maintained stoichiometric stability under different P addition treatments (Han et al., 2014).

Our results suggest that P enrichment decreased foliar N content and increased C:N, which is consistent with the results of other studies (Feller et al., 2007; Yuan and Chen, 2015). The main reason for this finding might be that increased P availability generally stimulates plant growth in P-limited ecosystems, leading to a decline in plant N concentration owing to the dilution effect (Yuan and Chen, 2015; Mao et al., 2016). Another possible reason might be the decrease in soil total N content in the one–time P addition treatment (Table 1). He and Dijkstra (2015) also confirmed that P addition can result in considerable losses of gaseous N from P-poor soils, most likely via direct stimulation of nitrification and denitrification. However, our results differ from those of some other studies (Newman et al., 2004; Mao et al., 2016). In a northern Everglades slough wetland, N concentrations in water lily generally increased in response to increased P loads (Newman et al., 2004), which might have been due to increased soil pore water NH_4_-N concentrations, mainly from the increased decomposition of resultant nutrient regeneration (Newman et al., 2001). These results suggested that the influence of P enrichment on plant stoichiometry may vary with wetland type and is possibly related to nutrient-limited conditions (Mao et al., 2016).

In conclusion, our results showed that P enrichment promoted plant growth and that this effect increased with decreasing addition frequency. Moreover, we also confirmed that P enrichment, irrespective of the frequency, increased plant C:N and P concentration, but decreased plant N concentration, C:N, and N:P. In recent years, increasing amounts of P have been discharged into Dongting Lake, due to the high intensity of anthropogenic activities. Our results improve our understanding of the influence of P enrichment on wetland nutrient cycling in this lake. However, P input usually occurs with other nutrient elements e.g., N and K. Therefore, the influences of other elements, as well as their interactive effects on plant stoichiometry should be studied to better understand the influence of exogenous nutrient input on nutrient cycling in Dongting Lake.

## Author Contributions

FL and CH wrote the manuscript and conducted the technical assays and statistical analysis. YX and WL designed the experiment and edited the manuscript. FL, CH, XC, ZD, and ZH contributed to data collection and interpretation. All authors reviewed the manuscript.

## Conflict of Interest Statement

The authors declare that the research was conducted in the absence of any commercial or financial relationships that could be construed as a potential conflict of interest.
